# Chronic Prostatitis/Chronic Pelvic Pain Syndrome Induces Depression-Like Behavior and Learning-Memory Impairment: A Possible Link with Decreased Hippocampal Neurogenesis and Astrocyte Activation

**DOI:** 10.1155/2023/3199988

**Published:** 2023-04-06

**Authors:** Nikola Šutulović, Milena Vesković, Nela Puškaš, Aleksa Zubelić, Djurdja Jerotić, Sonja Šuvakov, Sanja Despotović, Željko Grubač, Dušan Mladenović, Djuro Macut, Aleksandra Rašić-Marković, Tatjana Simić, Olivera Stanojlović, Dragan Hrnčić

**Affiliations:** ^1^Institute of Medical Physiology “Richard Burian”, Belgrade University Faculty of Medicine, 11000 Belgrade, Serbia; ^2^Institute of Pathophysiology “Ljubodrag Buba Mihailovic”, Belgrade University Faculty of Medicine, 11000 Belgrade, Serbia; ^3^Institute of Histology and Embryology “Aleksandar Đ. Kostić”, Belgrade University Faculty of Medicine, 11000 Belgrade, Serbia; ^4^Institute of Clinical and Medical Biochemistry, Belgrade University Faculty of Medicine, 11000 Belgrade, Serbia; ^5^Division of Nephrology and Hypertension, Mayo Clinic, Rochester, MN 55902, USA; ^6^Clinic of Endocrinology, Diabetes and Metabolic Disease, University Clinical Centre of Serbia, Belgrade University Faculty of Medicine, 11000 Belgrade, Serbia

## Abstract

Pathogenesis of chronic prostatitis/chronic pelvic pain syndrome (CP/CPPS) remains unclear since it represents an interplay between immunological, endocrine, and neuropsychiatric factors. Patients suffering from CP/CPPS often develop mental health-related disorders such as anxiety, depression, or cognitive impairment. The aim of this study was to investigate depression-like behavior, learning, and memory processes in a rat model of CP/CPPS and to determine the alterations in hippocampal structure and function. Adult male Wistar albino rats (*n* = 6 in each group) from CP/CPPS (single intraprostatic injection of 3% *λ*-carrageenan, day 0) and Sham (0.9% NaCl) groups were subjected to pain threshold test (days 2, 3, and 7), depression-like behavior, and learning-memory tests (both on day 7). Decreased pain threshold in the scrotal region and histopathological presence of necrosis and inflammatory infiltrate in prostatic tissue confirmed the development of CP/CPPS. The forced swimming test revealed the depression-like behavior evident through increased floating time, while the modified elevated plus maze test revealed learning and memory impairment through prolonged transfer latency in the CP/CPPS group in comparison with Sham (*p* < 0.001 and *p* < 0.001, respectively). Biochemical analysis showed decreased serum levels of testosterone in CP/CPPS group vs. the Sham (*p* < 0.001). The CP/CPPS induced a significant upregulation of ICAM-1 in rat cortex (*p* < 0.05) and thalamus (*p* < 0.01) and increased GFAP expression in the hippocampal astrocytes (*p* < 0.01) vs. Sham, suggesting subsequent neuroinflammation and astrocytosis. Moreover, a significantly decreased number of DCX+ and Ki67+ neurons in the hippocampus was observed in the CP/CPPS group (*p* < 0.05) vs. Sham, indicating decreased neurogenesis and neuronal proliferation. Taken together, our data indicates that CP/CPPS induces depression-like behavior and cognitive declines that are at least partly mediated by neuroinflammation and decreased neurogenesis accompanied by astrocyte activation.

## 1. Introduction

One of the most common urological conditions in the men population under the age of 50 is chronic prostatitis/chronic pelvic pain syndrome (CP/CPPS, type III prostatitis per NIH classification). This form of prostatitis is clinically manifested with pain in the pelvic region, rectum, lower abdomen, and testicles, usually accompanied by urogenital dysfunction including ejaculation and urination problems [1–3]. The pathogenesis of CP/CPPS is complex and actually represents an interplay between immunological, endocrine, and neuropsychiatric factors, and therefore, it remains unclear in contrast to prostatitis type I and II caused by bacterial infections. Inflammation is a start point for the pathogenesis investigation, and numerous studies confirmed increased concentrations of tumor necrosis factor *α* (TNF-*α*) and interleukin- (IL-) 1*β* in seminal fluid in men [4, 5]. Another important factor in prostatitis pathogenesis is sex hormones, predominantly testosterone which is shown to exert a protective role in prostate inflammatory diseases in human studies, but its concentrations are decreased in patients with prostatitis [6]. Besides, animal experiments also showed that the administration of testosterone prevented estrogen-induced prostatitis [7]. The presence of pain indicates the involvement of neuronal factors and the presence of neuroinflammation in the peripheral and central nervous system (CNS) [8]. We have previously reported increased levels of IL-1*β* and IL-6 in the cortex and thalamus in rats with CP/CPPS which confirmed the presence of neuroinflammation in the CNS in our animal model [9].

It is not rare that in patients suffering from CP/CPPS psychological and psychosocial disorders may occur. Various studies confirm the structural and functional changes in the CNS in patients with CP/CPPS, as well as the link between CP/CPPS and psychiatric conditions such as anxiety and depression [10–13]. Depression represents a wide spectrum of psychiatric disorders that includes long-lasting sadness, worthlessness, lack of motivation, and decreased self-confidence and self-esteem [14]. Piontek et al. [15] observed depression behavioral patterns in patients with CP/CPPS, especially during the exacerbation of the disease. Besides emotional disturbances, patients with CP/CPPS suffer from cognitive deficits [16, 17]. Impairment in learning and memory can negatively affect patients' quality of life. Recent studies are providing new shreds of evidence to prove the connection between learning-memory declines and CP/CPPS, but still, precise pathophysiological mechanisms are not completely revealed. Functional and structural changes in various brain structures (cortex, thalamus, hippocampus, and others) can be associated with the development of CP/CPPS but could also be a consequence of CP/CPPS [18]. One of the main mechanisms connecting CP/CPPS with psychosocial disorders could be related to the hypothalamic-pituitary-adrenal (HPA) axis dysfunction. Besides, neuropathic pain that occurs in CP/CPPS is supposed to be linked with activation of the HPA axis and elevated corticosterone affecting significantly animal behavior [19]. Among brain structures, the hippocampus has an important role in the HPA axis regulation, representing a key point in stress, pain, and emotional response. Many psychiatric disorders are linked to dysfunction in hippocampal inhibitory neurotransmission [20–22]. Our previous studies investigating mental health disorders related to CP/CPPS suggest that proinflammatory mediators derived from prostate can cross the brain-blood barrier and accumulate in several brain areas [9, 13]. In addition, this inflammation in CNS is considered to be responsible for the depression development [23]. Furthermore, cytokines can activate glial cells in CNS, affecting synaptic density and plasticity and contributing to the development of depression [24, 25]. In recent years, more attention is given to the role of astrocyte activation in the pathogenesis of cognitive disorders as well. It has been shown that astrocytes, as important immune cells in the CNS, may play an important role in hippocampus-dependent cognitive impairment in CP/CPPS [26].

However, so far, there have been no studies that examined combined depression and cognitive disorders in the model of CP/CPPS. Therefore, this study was aimed at investigating depression-like behavior, learning, and memory processes in a rat model of CP/CPPS, from the aspect of indicators of neuroinflammation, neurogenesis, and astrocytosis.

## 2. Materials and Methods

### 2.1. Ethical Permissions and Directives

We conducted all our experimental treatments and techniques in full compliance with the Directive of the European Parliament and the Council (2010/63/EU). Also, we obtained permission (No. 323-07-01339/2017-05/3) for our study from the competent ethical body of Belgrade University School of Medicine (Animal Welfare Committee).

### 2.2. Animals and Housing

For our experiments, we used 90-day-old 12 male *Wistar albino* rats with a mass of 250–350 g at the start of research. Animals were delivered from accredited breeding laboratory (Military Medical Academy, Belgrade, Serbia) and kept in standard housing cages with unlimited access to standard food and water. In the course of preexperimental 7-day acclimation period and during the experiment, animal room environmental parameters were controlled (room temperature was 22-24°C, relative air humidity was 50 ± 5%, and the light phase of light : dark cycle started on 08:00 a.m. and ended on 08:00 p.m.).

### 2.3. Experimental Protocol

**Figure 1:**
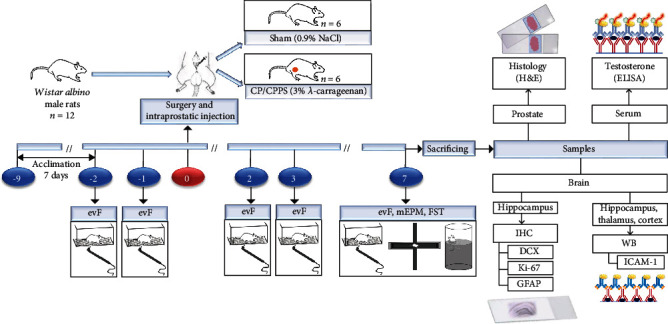
Time course of experimental protocol. Acclimation of rats to laboratory conditions started nine days prior to intraprostatic injection (-9). On day 0 of the experiment, surgery was performed, and 12 rats were assessed randomly into the Sham group (injection of sterile saline into prostate; *n* = 6) and the CP/CPPS group (injection of 3% *λ*-carrageenan into prostate; *n* = 6). Pain thresholds were assessed using evF before (days -2 and -1) and after (days 2, 3, and 7) surgery. Cognitive functions and depression-like behavior were estimated 7^th^ upon operation by mEPM test and FST. After test completion (day 7), rats were sacrificed and sera were isolated from blood for testosterone concentration assessment by ELISA, while the ICAM-1 expression in hippocampi, thalami, and cortices is determined by WB. After brain slice preparation and IHC staining, hippocampal expression of DCX and Ki-67 and GFAP representation were quantified. evF: electronic von Frey aesthesiometer; mEPM: modified elevated plus maze test; FST: forced swimming test; ELISA: enzyme-linked immunosorbent assay; ICAM-1: intercellular adhesion molecule 1; WB: Western blot; IHC: immunohistochemistry; DCX: doublecortin; GFAP: glial fibrillary acidic protein.

In line with our previous data and literature protocols [9, 13, 27, 28], we randomly divided rats (day 0), into two groups. First was the sham-surgery control group in which we injected 50 *μ*l of sterile saline in both ventral prostate lobes (Sham, *n* = 6), and the other one was the experimental group in which we treated rats with 3% *λ*-carrageenan by the same way (CP/CPPS, *n* = 6). To verify CP/CPPS development, pain thresholds on mechanical stimuli were assessed in the skin of the scrotum by an electronic von Frey aesthesiometer (evF) in basal conditions, 2 consecutive days prior to intraprostatic injection (days -2 and -1), as well as in the 2^nd^, 3^rd^, and 7^th^ postoperative days (days 2, 3, and 7). To determine cognitive functions and depression-like behavior, the 7^th^ day after intraprostatic injection, animals from both groups (*n* = 6 in each group) were tested in modified elevated plus maze test (mEPM) and forced swimming test (FST). Just after the termination of the tests, all animals were sacrificed to collect blood, brain, and prostate tissues in the purpose for histological examination, biochemical analyses, and immunohistochemical staining. Blood was collected for sera isolation and testosterone concentration measurement. Brains were collected to determine ICAM-1 expression in isolated brain structures, while in hippocampi we estimated the expression of DCX and Ki67, as well as the representation of GFAP+ cells. Prostates were stained by hematoxylin-eosin (H&E) technique and examined microscopically. In Figure 1, we schematically presented the time course of all our experimental procedures.

### 2.4. Induction of Experimental CP/CPPS

Experimental CP/CPPS in our current study was induced on the same way as we described in detail in our previous studies [9, 13]. Briefly, procedure consisted of rat anesthetization, preparation of surgery field of the lower abdomen, rats fixation, an abdominal incision, exposition of left and right ventral prostate lobes, 50 *μ*l sterile 3% *λ*-carrageenan solution (Sigma Aldrich, St. Louis, MO, USA) or sterile saline injection in prostate, and wound suturing.

### 2.5. Assessment of Pelvic Pain Threshold

A platform for scrotal pain threshold assessment consisted of plexiglass cubicles with a wired bottom and eVF (IITC Life Sciences, CA). Scrotal skin pain threshold measurement was performed in accordance with the protocol described in detail in our previous studies [9, 13]. Briefly, it consisted of 30 minutes of rat's adaption to the platform and gradual evF filament application until observation of rat's withdrawal reflex (a sudden move from the initial position). The values of pain threshold were calculated as the average value of 3 repeated measurements.

### 2.6. Histological Examination of Prostates

Instantly upon sacrifice, prostates were isolated from all rats and processed by standard H&Е technique which we described in previous research [9, 13]. Slices were examined under the Leica DM4000 B LED microscope, while microphotographs were obtained by digital camera Leica DFC295 using Leica Application Suite (LAS, v4.4.0) software package.

### 2.7. Depression-Like Behavior Assessment

According to the previously described methodology by Porsolt et al. [29], we performed the forced swimming test (FST). Rats were immersed individually in plastic cylinders (50 cm height with inner diameter of 25 cm) filled by 22 ± 1°C tap water to the 35 cm height, following 5 minutes video monitoring of behavior. Three different behavioral patterns, observed during the FST, are considered: (a) struggling (climbing): active movements of all four extremities with the front limbs thrashing against walls or to hit the surface of the water; (b) swimming (active swimming): diving or movements across the water which are parallel to surface; and (c) floating (passive swimming, immobilization): floating horizontally on the water surface, with movements just to maintain the nose and/or part of the head above the water surface. Upon the test completion, rats were carefully moved out from the cylinders, dried, and placed to original cages. Behavioral patterns during the five-minute FST were later carefully analyzed from the obtained video records, and the distribution of struggling, swimming, and floating was estimated. To avoid mistaken results, the animals were adapted (pretested) to FST for 15 minutes, two consecutive days before the test.

### 2.8. Cognitive Behavior Assessment

To test cognitive behavior, we used modified elevated plus maze test which is described in the literature [30]. A modified platform of elevated plus maze (Elunit, Belgrade, Serbia), which was placed 50 cm above the floor, consisted of two enclosed and two open arms emerging from a central square (10 × 10 cm) in the direction opposite to each other. The test is created on the basis of innate antipathy of rats to bright and unprotected spaces (analogous to open arms) and the natural tendency to find a safe shelter (analogous to closed arms). Thus, cognitive behavior, learning, and memory are reflected in the time that animals take to remember and learn the arrangement of open and closed arms. The test was performed on two consecutive days. During the first day of the test (pretest session), each animal was positioned individually at the open arm end which is opposite to the central platform, and the time which is needed to rat make a move to the left or right closed arm (transfer latency) was recorded (T1). The maximal time which was allowed to an animal to explore the platform and find the closed arm was 90 seconds. If rat unsuccessfully found closed arm for 90 seconds, it was allowed to place the rat in the closed arm and permit them to investigate it for additional 60 seconds, and the time of 90 seconds was taken for the value of T1. After a period of 24 hours, the procedure was repeated in the same way (retention session), and the transfer latency (T2) was recorded. It is expected that repeated exposure of animals shortens transfer latency, so the value of T2 was used as the parameter of cognitive abilities, learning, and memory. Impaired cognitive behavior is reflected in the increase of T2. All test sessions were monitored and recorded with an infrared camera (HikVision Bullet 2612, Hangzhou, China) connected to a computer and placed above the platform. Between two animal testing, the platform was cleaned to remove olfactory stimuli. Offline analysis was carefully performed from the obtained recordings by the same blinded experimenter.

### 2.9. ELISA for Testosterone

With the purpose of minimalizing the effect of circadian alteration in testosterone secretion [31], blood samples from all 12 rats (*n* = 6 in each group) were taken the 7^th^ postoperative day at 9:00-10:00 a.m. using blood collecting equipment. After blood sampling, sera were isolated by our standard, previously described methodology [13]. Serum testosterone levels were determined by commercially available Rat Testosterone (*T*) ELISA Kit (Cusabio, Houston, USA) by following the manufacturer's recommendations. The minimal concentration which ELISA kit was able to detect was <0.06 ng/ml. The standard curve ranged from 0.13 to 25.6 ng/ml. To calculate *T* concentrations, we created a standard curve using “Curve Expert 1.4”. Duplicate serum aliquots were used.

### 2.10. Western Blotting

The seventh day upon intraprostatic injection, after the test completion, rats from both groups were sacrificed and brains were isolated from the skull. Randomly and alternately, we used one of the brain hemispheres to assess brain ICAM-1 expression (*n* = 4 in each group), while another one (*n* = 6 in each group) is processed in IHC analysis. Hippocampi, cortices, and thalami were isolated and underwent homogenization in RIPA buffer by a standard, previously described procedure [13]. Protein concentrations were determined by the Bicinchoninic Acid (BCA) Protein Assay (Sigma, Germany). Western blot was performed according to our previously described technique [32]. Briefly, a total of 30 *μ*g of proteins were loaded on Criterion™ TGX precast 26-well gel (4-15%) (Bio-Rad, USA) before electrophoresis performing which was followed by 1 hour long (at room temperature) and overnight (at 4°C) incubation with primary monoclonal mouse anti-ICAM1 (1: 200, Santa Cruz, USA) and monoclonal mouse anti-*β* actin antibodies (1 : 10 000, Thermo Fisher Scientific, UK). Next day, membrane was washed in TBS-Tween and incubated with secondary anti-mouse antibody (1 : 4000, Abcam, UK) for 1 hour (at room temperature). Chemiluminescent bands were identified using the Clarity™ Western ECL Substrate (Bio-Rad, USA) on a chemiluminescence reader. Analysis of densitometry was done using ImageJ software package (National Institutes of Health, Bethesda, USA).

### 2.11. Immunohistochemical Staining and Quantification

Randomly, the left or right brain hemisphere from all rats (*n* = 6 in each group) was processed in IHC staining by a standard procedure which we in detail described in our previous research [13]. After tissue fixation and paraffin embedding by standard procedure, coronal 4 *μ*m thick sections were cut, dried, and then dewaxed in xylene, rehydrated, and treated with citrate buffer. Activity of endogenous peroxidase was blocked with 3% hydrogen peroxide, while 1-hour incubation with normal rabbit (for DCX) or goat (for Ki-67 and GFAP) serum was used to block nonspecific labeling. Thereafter, slices were incubated overnight at room temperature with the following primary antibodies: goat polyclonal anti-DCX (1 : 100, #sc-8066, Santa Cruz Biotechnology, USA), rabbit polyclonal anti-Ki-67 (1 : 3500, #ab15580, Abcam, United Kingdom), and rabbit polyclonal anti-GFAP (1 : 500, #z0334, DAKO, Santa Klara, USA). Labeling was performed by 1-hour incubation with the following biotinylated secondary antibodies: rabbit anti-goat antibody for DCX (VECTASTAIN® Elite ABC-HRP Kit, #PK-6105, Vector Laboratories, USA) and goat anti-rabbit antibody for Ki-67 and GFAP (VECTASTAIN® Elite ABC-HRP Kit, #PK-6101, Vector Laboratories, USA), which was followed by avidin-biotin-horseradish peroxidase complex (Vector Laboratories, USA). Between all the mentioned steps, the slices were washed 3 times for 5 minutes in PBS (pH = 7.4). Sites of immunoreactivity was visualized using 3,3′-diaminobenzidine chromogens (DAB Peroxidase Substrate Kit, SK-4100, Vector Laboratories, USA). At the end of staining, Mayer's hematoxylin was used to slice counterstaining. Finally, after dehydration in ethanol, slices are protected by DPX mounting medium (Sigma-Aldrich, USA).

Analysis of immunohistochemical slices and immunoreactivity were performed on a microscope with a digital camera which we mentioned above. Estimation of DCX, Ki-67, and GFAP immunoreactivity was performed in all rat hippocampi (*n* = 6 in each group). Quantification of DCX+ and Ki67+ neurons was performed along the subgranular zone (SGZ) of the dentate gyrus (DG) of the hippocampus, which is, in accordance with the literature data [33], defined as the boundary between the DG hilus and the granular cell layer. The length of the SGZ was measured using the Leica Application Suite (LAS, v4.4.0) software package, and regardless of size or shape, DCX and Ki67-immunoreactive neurons were counted. To standardize the number of cells (different length of SGZ), the number of DCX+ and Ki67+ cells was expressed per 1 mm of SGZ length. A representation of GFAP-immunoreactivity was assessed in the DG of the hippocampus using the Color Picker Threshold Plugin as part of the Icy software package (Pasteur Institute and France BioImaging National Coordination Team, France). The first step was to mark positive colors (several shades of brown color representing GFAP-immunoreactive cells stained with DAB) and negative colors (blue, white and light brown colors representing the nuclei of cells that are contrasted with hematoxylin, discolored tissue and bright, nonselective stained background) using the “Color Picker Threshold Plugin”. Based on the correct selection of positive and negative colors, the software package measured the surface covered with positive colors (surface of GFAP-immunoreactivity). The representation of GFAP-immunoreactive cells is expressed by the relative ratio between the surface of the GFAP-immunoreactive cells and the total area of the region of interest (hippocampal DG) in percent (%).

### 2.12. Data Processing

To test the normality of variables distribution, the Kolmogorov-Smirnov test was used. The normal distribution of data was demonstrated in all output data. So, we expressed all our parameter values as means ± standard deviation (SD). To test the statistical significance of differences between CP/CPPS and Sham groups, we performed Student's *t*-test. To test statistical significance of within-group differences in different time points, we used the analysis of variance (ANOVA, one way) with the Tukey-Kramer LSD post hoc test. The levels of statistical difference were *p* < 0.05, *p* < 0.01, or *p* < 0.001.

## 3. Results

### 3.1. The Animals with CP/CPPS Exhibit Lower Scrotal Pain Thresholds, Prostate Inflammation, Depression-Like Behavior, and Impaired Cognitive Functions

**Figure 2:**
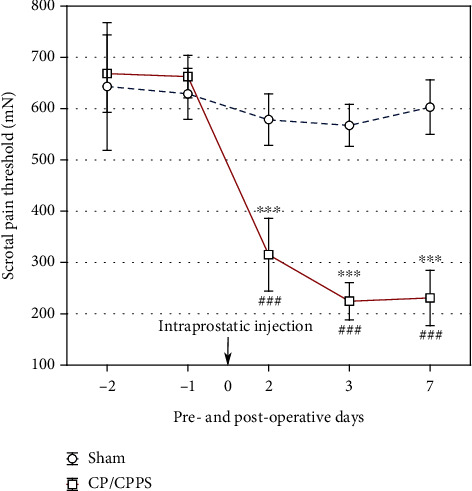
Pelvic pain thresholds in preoperative and postoperative days. Pain thresholds were assessed in the scrotal skin by evF in two consecutive days before (-2 and -1), together with days after (2, 3, and 7) operation and intraprostatic injection (0). Pain thresholds are expressed as the mean ± SD. Between-group differences were assessed by Student's *t*-test (^∗∗∗^*p* < 0.001, *vs.* Sham, *n* = 6 in each group), while within-group differences were assessed using analysis of variance (ANOVA, one way) with the Tukey-Kramer LSD post hoc test (^###^*p* < 0.001, *vs.* -1, *n* = 6 per group).

There were no statistically significant differences in pelvic pain thresholds between groups (Sham vs. CP/CPPS, *p* > 0.05, Figure 2) on the 2^nd^ and 1^st^ days before operation. Also, pelvic pain thresholds within the Sham animals showed no significant differences in all postoperative measurements (2^nd^, 3^rd^, and 7^th^ days), in comparison to the preoperative period (*vs.* -1, *p* > 0.05, Figure 2). CP/CPPS rats manifested significantly reduced (*p* < 0.001) scrotal skin pain thresholds in the all postoperative days (2^nd^, 3^rd^, and 7^th^ days), compared to the values in Sham rats (Figure 2). In addition, within-group distribution of pain thresholds dynamics in CP/CPPS group (Figure 2) showed significant reduction (*p* < 0.001) in all postoperative days, when compared to preoperative (*vs.* -1).

**Figure 3:**
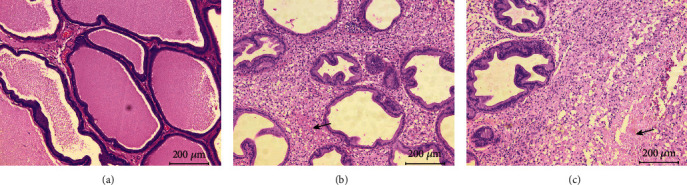
Representative histological images of rats' prostate. Unaltered histological prostate morphology in the Sham group (a). Interstitial proliferation with leukocyte infiltration and hyalinization (arrow) (b) or interstitial necrosis (arrow) (c) in the CP/CPPS group.

Prostates in rats injected with 0.9% NaCl manifested standard histological structure with the well-preserved structure (Figure 3(a)). On the other hand, prostates in CP/CPPS rats exhibited prostatic inflammation (Figures 3(b) and 3(c)). Signs of chronic prostate inflammation were the same in all CP/CPPS rats, and consisted of proliferation in interstitium, followed by leukocyte infiltration and hyalinization (arrow, Figure 3(b)) and interstitial necrosis (arrow, Figure 3(c)).

**Figure 4:**
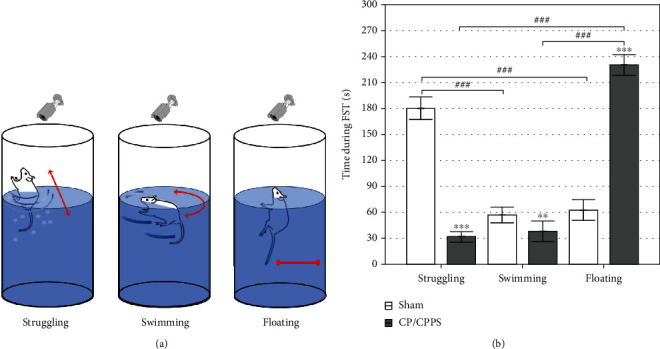
Graphic illustration (a) and distribution of different behavior patterns during FST (b). Time spent in struggling, swimming, and floating is expressed as the mean ± SD value. Between-group differences were assessed by Student's *t*-test (^∗∗∗^*p* < 0.001 vs. Sham, ^∗∗^*p* < 0.005 vs. Sham, *n* = 6 per group), while within-group differences were assessed using analysis of variance (ANOVA, one-way) with the Tukey-Kramer LSD post hoc test (^###^*p* < 0.001).

The illustration of three types of behavior during the FST is presented in Figure 4(a). Rats with experimentally induced CP/CPPS spent significantly less time struggling than Sham rats (*p* < 0.001; Figure 4(b)). The same holds for swimming (*p* < 0.005; Figure 4(b)), while CP/CPPS rats revealed significantly more time floating in comparison to the Sham rats (*p* < 0.001; Figure 4(b)). Within the group, FST behavioral pattern showed that in CP/CPPS rats predominantly observed behavior during the FST was floating in comparison to the struggling (*p* < 0.001; Figure 4(b)) and swimming (*p* < 0.001; Figure 4(b)), while in Sham rats it was struggling in comparison to the swimming (*p* < 0.001; Figure 4(b)) and floating (*p* < 0.001; Figure 4(b)).

**Figure 5:**
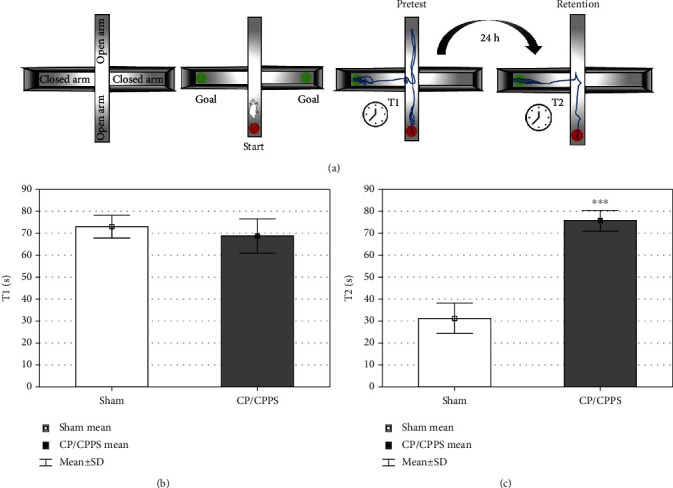
Schematic illustration (a) and transfer latency in pretest (b) and retention (c) during mEPM test. Transfer latency during pretest (T1) and retention (T2) are expressed as the mean ± SD value. Student's *t*-test was used to assess statistical significance of differences (^∗∗∗^*p* < 0.001 vs. Sham, *n* = 6 in each group).

Cognitive functions were tested in the mEPM test (Figure 5(a)). Six days after surgery, during the pretest, no significant differences (*p* > 0.05) were observed in the duration of transfer latency (T1) between CP/CPPS and Sham rats (Figure 5(b)). One day later, on the 7^th^ postoperative day, the retention session was conducted. We observed significantly prolonged transfer latency (T2) in CP/CPPS rats (*p* < 0.05) in comparison compared to the corresponding controls (Figure 5(c)).

### 3.2. Experimental CP/CPPS Led to Reduced Serum Testosterone Level and Increased ICAM-1 Expression in Brain Structures

**Figure 6:**
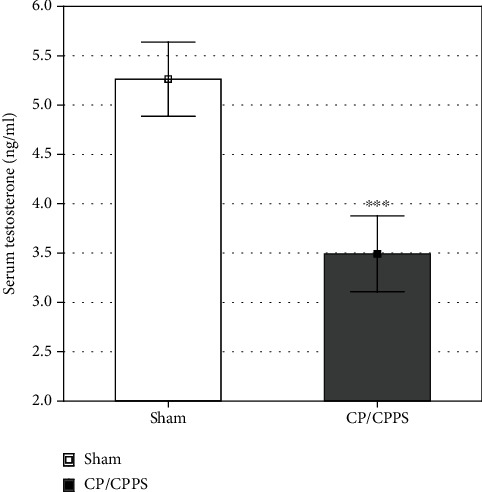
Testosterone concentration in serum of Sham and CP/CPPS rats. ELISA was used to determine the concentrations of testosterone. Data are the means ± SD values. Student's t-test was used to assess statistical significance of differences (^∗∗∗^*p* < 0.001 vs. Sham, *n* = 6 in each group).

Serum testosterone concentrations in rats with experimentally induced CP/CPPS showed a statistically significant decrease in comparison to the corresponding controls (*p* < 0.001; Figure 6).

**Figure 7:**
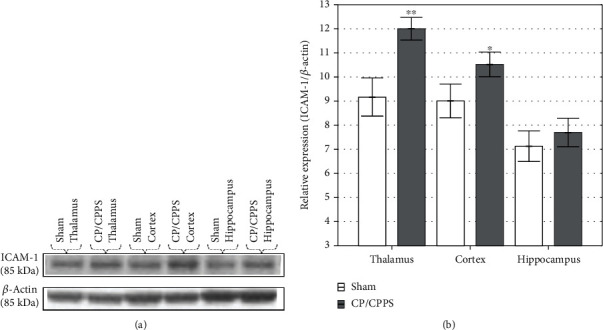
Hippocampal ICAM-1 expression. Blots denote nonadjacent bands from the same gel (a). ICAM-1 protein expression in the cortices and thalami of CP/CPPS rats was significantly increased in the comparison to the Sham rats (b). Data are the means ± SD values. Student's *t*-test was used to assess statistical significance (^∗∗^*p* < 0.01 and^∗^*p* < 0.05 vs. Sham, *n* = 4 in each group).

A region-specific increase in ICAM-1 expression was observed using Western blot analysis. Rats from the CP/CPPS group showed a nonsignificant increase of ICAM-1 protein expression in the hippocampi when compared to the controls (*p* > 0.05; Figure 7). The same alteration was observed regarding the expression of the ICAM-1 protein in the cortices, with a significantly higher expression in the CP/CPPS group when compared to the Sham group (*p* < 0.05; Figure 7). Also, CP/CPPS rats revealed a statistically significant increase in thalamic ICAM-1 protein expression in comparison to Sham rats (*p* < 0.05; Figure 7).

### 3.3. Experimental CP/CPPS Is Associated with Decreased Hippocampal Neurogenesis and Hippocampal Astrocytosis

**Figure 8:**
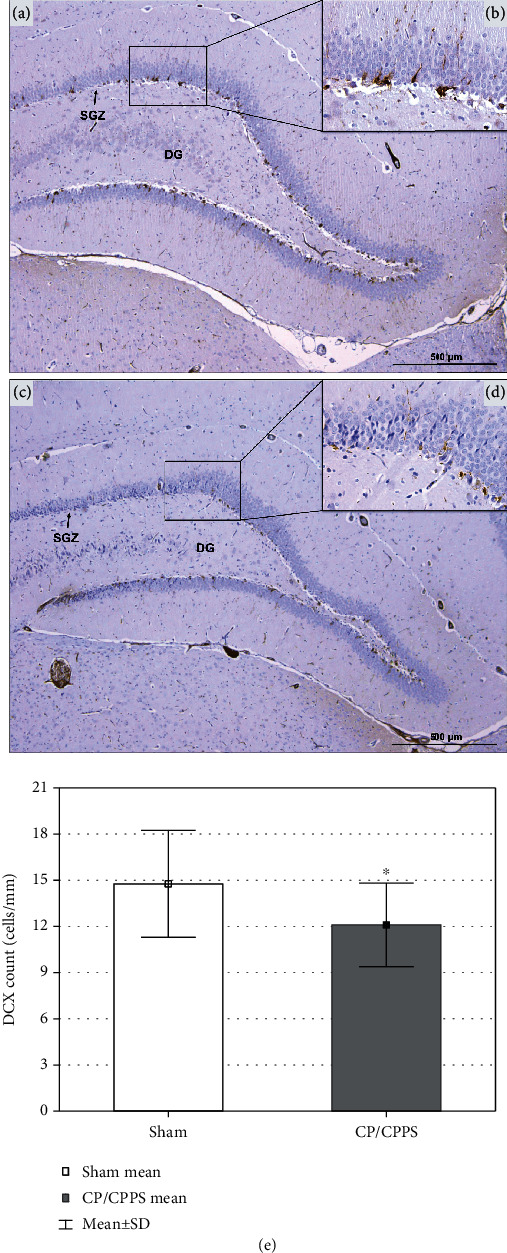
Hippocampal DCX immunohistochemical expression. Representative photomicrography of rat hippocampal immunohistochemical expression of DCX in Sham (a) and CP/CPPS (c) rats with labeled area of subgranular zone (SGZ) in dentate gyrus (DG). An enlarged image of few DCX+neurons of Sham (b) and CP/CPPS (d) rats mainly located in the SGZ of DG. The number of DCX+ cells in hippocampi of both animal groups (e) is expressed per 1 mm of the SGZ length. Data are the means ± SD. Student's *t*-test was used to assess statistical significance (^∗^*p* < 0.05 vs. Sham).

DCX+ neurons in the investigated groups were located mostly in the area of the SGZ of the hippocampal DG (Figures 8(a)–8(d)). On the other hand, quantification of DCX+ neurons in the SGZ showed significantly lower number in CP/CPPS rats, comparing to Sham (*p* < 0.05, Figure 8(e)).

**Figure 9:**
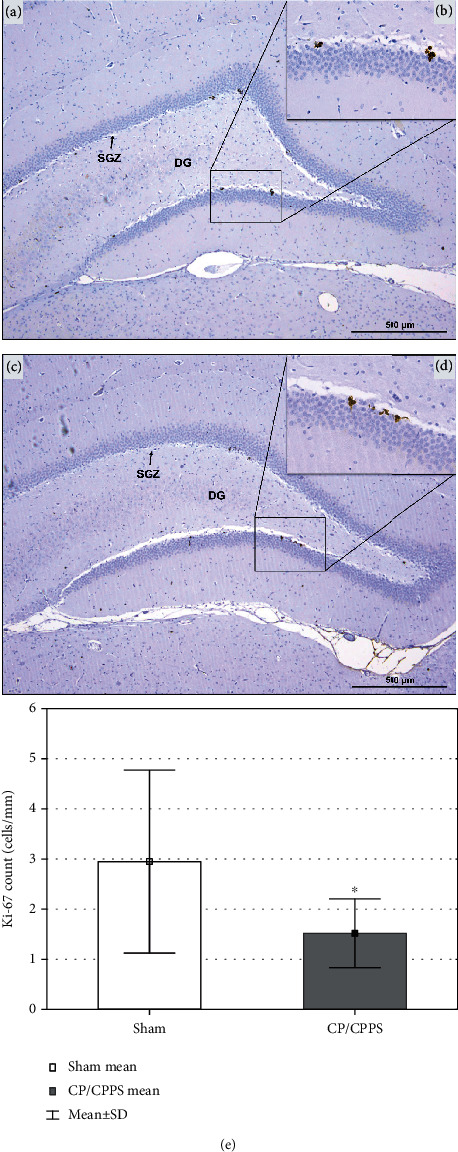
Hippocampal Ki-67 immunohistochemical expression. Representative photomicrography of rat hippocampal immunohistochemical expression of Ki-67 in Sham (a) and CP/CPPS (c) rats with labeled area of subgranular zone (SGZ) in dentate gyrus (DG) (a). Enlarged image of few Ki-67+ neurons of Sham (b) and CP/CPPS (d) rats mainly located in the SGZ of DG (b). The number of Ki-67+ cells in hippocampi of both animal groups (e) is expressed per 1 mm of the SGZ length. Data are the means ± SD. Student's *t*-test was used to assess statistical significance (^∗^*p* < 0.05 vs. Sham).

Ki-67+ neurons in rats from both groups were widely visualized within the SGZ in the DG of the hippocampus (Figures 9(a)–9(d)). Further Ki-67 protein expression quantification in hippocampi of these rats showed significant difference between groups. As shown in Figure 9(e), the number of Ki-67+ neurons in SGZ of the hippocampal DG in CP/CPPS rats was significantly less, in comparison to Sham rats (*p* < 0.05).

**Figure 10:**
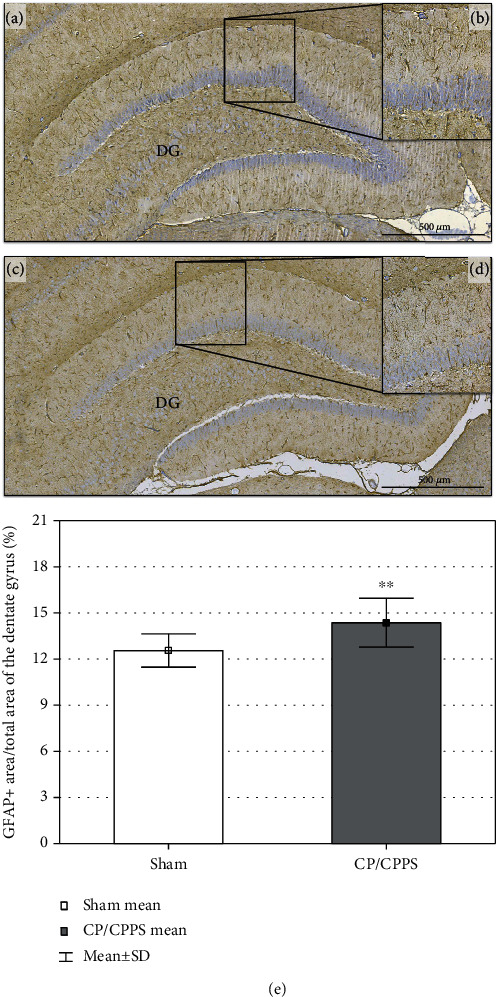
GFAP hippocampal immunohistochemical representation. Representative photomicrography of hippocampal GFAP immunohistochemical representation of labeled area of dentate gyrus (DG) in Sham (a) and CP/CPPS groups (c). Enlarged image of GFAP+ cells in Sham (b) and CP/CPPS (d) rats located in the granular cell layer of DG. Representation of GFAP+ cells in DG of hippocampi of both animal groups (e) is expressed as area of GFAP+ cells divided with total area of DG in percent (%). Data are the means ± SD. Student's *t*-test was used to assess statistical significance (^∗∗^*p* < 0.01 vs. Sham).

In our study, we also analyzed astrocytes from the granular cell layer within the DG of the hippocampus. In both groups, the GFAP+ astrocytes soma and processes were observed on immunohistochemical images (Figures 10(a)–10(d)). Quantitative analysis was done by the determination of the representation of GFAP+ cells in DG of hippocampi, expressed as the area of GFAP+ cells divided by total area of DG in percent (%). We observed an increased representation of GFAP+ cells in the CP/CPPS group (*p* < 0.01) when compared to corresponding controls (Figure 10(e)).

## 4. Discussion

Our results indicate that CP/CPPS induces a significant decrease in the pain threshold for mechanical stimuli on scrotal region skin in comparison with the control group (Figure 2). Histological analysis of prostate tissue reveals interstitial proliferation with leukocyte infiltration and hyalinization followed by focal interstitial necrosis in CP/CPPS groups (Figure 3), confirming the inflammatory process and the development of prostatitis with pain syndrome. Critically, our results indicate the presence of mood disorder in the CP/CPPS model. Forced swimming test showed that animals with CP/CPPS exert depression-like behavior (Figure 4). In addition, these animals also presented impaired cognitive functions, learning, and memory, confirmed by modified elevated plus maze test (Figure 5). Clinical studies also confirmed that patients with CP/CPPS often may have mental health problems and mood disorders. A cohort study by Clemens et al., conducted in 2008, in the United States [34], showed that approximately 18% of patients with CP/CPPS also use some type of antidepressants or anxiolytics due to psychiatric comorbidities. Similar studies have indicated that the negative impact of CP/CPPS on quality of life and mental health is significantly higher than in other diseases of the genitourinary tract and can be compared with conditions such as coronary heart disease [35]. Besides, one of our recent studies showed increased anxiety-linked behavior in rats with CP/CPPS indicating an interplay between brain oxidative distress, HPA activation, and decrease of hippocampal inhibitory interneurons [13]. On the other hand, not so many human or animal studies have evaluated cognitive impairment in CP/CPPS. By using the Morris water maze, Du et al. [36] have shown a significant decrease in spatial learning and memory in mice with experimental autoimmune prostatitis. Our present study is the first that evaluates both, depression-like behavior and cognitive performance, in a rat model of CP/CPPS to show close relationship between these disorders and to possibly explain mutual pathophysiological mechanisms involved in their pathogenesis.

There are several potential mechanisms that could explain how a peripheral inflammatory process, such as prostatitis, can lead to behavioral changes, depression, and cognitive impairment. The most accepted theories are based on inflammation, neurotransmission disorders, and dysfunction of the HPA axis [25]. A clinical study by Lee and Lee [37] found a correlation between CP/CPPS, particularly prostatitis-like symptoms and low serum total testosterone concentration. Low testosterone levels were also registered in rat's serum in our study after inducing prostatitis (Figure 6). There are evidence suggesting that CP/CPPS is related to systemic inflammation as in similar neuropathic pain conditions [38], while studies regarding prostatitis-induced pain syndrome pointed at the role of low testosterone level in systemic inflammation. It has been shown that exogenous androgens exert anti-inflammatory effect and that hypogonadism is associated with increased inflammatory markers [39], giving us more evidence of low testosterone level role in systemic inflammation. Systemic inflammation is closely connected with neuropsychiatric manifestation of chronic systemic inflammation and chronic pain syndrome [40]. An association between elevated levels of proinflammatory cytokines and the development of psychiatric disorders, such as depression, was first observed in hepatitis C patients treated with interferon gamma (IFN-*γ*) for therapeutic purposes [41]. On the other hand, the analysis of peripheral blood of patients, with clinically verified depression, confirmed the existence of elevated values of inflammatory markers [42]. The CNS is not an immunoprivileged organ because peripheral inflammatory processes can be followed by penetration of proinflammatory cytokines from the periphery into the CNS through the disrupted blood-brain barrier, consequently leading to the glial cell activation and neuroinflammation [9, 43]. We have previously shown that proinflammatory cytokines IL-6 and IL-1*β* were significantly increased in prostate tissue as well as in cortex and thalamus in rats with experimental CP/CPPS [9]. Similarly, Du et al. [25] showed the increase in hippocampal inflammatory mediators (IL-1*β*, IL-6, IL-8, and TNF-*α*) in mice with experimental autoimmune prostatitis. However, brain regions are specifically affected by neuroinflammatory processes that are caused by inflammation of peripheral organs. Due to diversity of glial and neuronal cells in various brain regions, it has been shown that there are regional differences in cytokine release and inflammation [44]. Results from our previous research on the same CP/CPPS rat model indicated an increase in IL-1 and IL-6 in cortex and thalamus, but not in the hippocampus [9]. Since it is known that these cytokines promote ICAM-1 expression, this may be the explanation for increased level of ICAM-1 in thalamus and cortex, but not in the hippocampus (Figure 7) in the present study. Since ICAM-1 represents an adhesion receptor that promotes leukocyte migration from circulation to inflammation site [45], our results may confirm the presence of inflammation in brain regions, possibly contributing to cognitive and behavioral disorders. One of the potential pathogenic processes for prostatitis-induced depression and impaired cognition is that proinflammatory cytokines trigger neuroinflammation by activating astrocytes after crossing brain-blood barrier. Also, synaptic changes caused by astrocytosis and astrocyte-derived proinflammatory mediators in the hippocampus may underlie the behavioral changes in existence of peripheral inflammation [46]. Recent studies support the fact that neuroinflammation plays a very important role in major depressive disorder [47]. Neuroinflammation followed by glial cell activation has been proposed by many clinical and preclinical studies as main pathophysiological process that underlies in the basis of various psychopathologies [48, 49]. Our results indicate increased activation of astrocytes in the hippocampus of rats with CP/CPPS which was evident by increased representation of GFAP+ cells (Figure 10). Astrocyte activation potentially may participate in CP/CPPS-induced memory and learning impairment by synaptic elimination through phagocytosis. Structural changes in synaptic plasticity and decreased dendritic complexity of hippocampal neurons were evident in mice model of experimental autoimmune prostatitis [25]. In the CNS, the hippocampus represents a very important brain region that is involved in the processes of learning, memory, navigation and social behavior [50]. Change in the astrocyte number in the hippocampus, together with increased astrocytes-derived proinflammatory cytokines level, is pointed as a contributing factor in depression pathogenesis [51]. Additionally, many animal studies showed that increased hippocampal astrocyte activation is linked with cognitive impairment [52]. Also, a study on a rodent model of prostatitis by Du et al. [25] linked depression-like behavior with changes in hippocampal astrocytes and microglial activation. Both glial markers Iba1 and GFAP were significantly overexpressed in the prostatitis group, and it was confirmed by ultrastructural changes in glial cells evident on electron microscopy. Another explanation from the same study is that activated microglia release proinflammatory cytokines changing the functional status of astrocytes [53]. Glial activation may affect the reuptake and release of glutamate, decreasing its level in the hippocampus and contributing to depression [25]. Studies using models of cerebrovascular and neurodegenerative diseases are also connecting glial activation and neuroinflammation with cognitive impairment. In cerebral hypoperfusion and Alzheimer's disease models, activated glia was shown to release proinflammatory factors such as IL-1*β*, IL-6, TNF-*α*, COX-2, and iNOS, promoting further neuronal degeneration in the hippocampus [54, 55]. Proinflammatory cytokines can exert inhibitory effects on the brain-derived neurotrophic factor- (BDNF-) signaling by activating p38MAPK and NF-*κ*B. Consequently, it reduces neurogenesis and neuronal plasticity [56, 57].

The effects of experimental CP/CPPS on the adult neurogenesis have not been the subject of research so far, and there are no data, in the available literature, on the direct influence of chronic prostatitis on neurogenesis, but more recent research has examined the role of decreased neurogenesis in mood disorders and cognitive decline.

Results of the present study showed a decreased number of DCX+ and Ki67+ neurons in hippocampi in CP/CPPS rats (Figures 8 and 9), suggesting that decreased neurogenesis and neuronal proliferation may underlie behavioral changes and cognitive impairment. Doublecortin, as the product of DCX gen, has a role in stabilization of microtubules and facilitation of their polymerization to promote the migration of postmitotic neurons. Actually, doublecortin, by acting via microtubules, modifies the cytoskeleton and helps neurons to migrate to a targeted location. Therefore, DCX is used as a marker of neuronal differentiation and migration in the assessment of the process of hippocampal neurogenesis [58].

Studies performed on mice have shown an increase in neurogenesis, as well as an increased density of the dendritic spine after the application of antidepressants or after voluntary running [59]. In a mouse model of emotional stress, Pavlov et al. [60] showed the presence of depression- and anxiety-like behavior in mice. Additionally, immunohistochemical staining revealed a decreased number of Ki67- and DCX-positive cells in the hippocampus of stressed animals, as well as increased markers of glia activation, which is in accordance with our findings. Cytokines, released from activated astrocytes, can further aggravate neuroinflammation and act inhibitory on neurogenesis. Their results confirmed increased expression of IL-1*β* and IL-6 in the hippocampus, which lead to decreased production of neuronal progenitors, BDNF, diminishing neurogenesis. Moreover, in a rat model of anxiety- and depression-like behavior induced by corticosterone, the number of DCX-positive cells in the hippocampus was significantly reduced. These data are consistent with our research supporting the theory that animals with experimental CP/CPPS may develop a disorder in neurogenesis due to peripheral inflammatory process, chronic stress, and pain, contributing further to neuropsychiatric comorbidities.

## 5. Conclusion

Our current study investigated, for the first time, both depression-like behavior and hippocampal-dependent cognitive performance and neurobiological factors in the hippocampus in a rat model of CP/CPPS. Based on our results, it may be suggested that both neuropsychiatric comorbidities that occurred in CP/CPPS may be, at least partly, attributed to the neuroinflammatory process and decreased neurogenesis mediated by astrocyte activation.

## Data Availability

The data used to support the findings of this study are available from the corresponding author upon reasonable request.
